# Cancers in DNA mismatch repair mutation carriers: results from a hospital based Lynch syndrome registry

**DOI:** 10.1186/1897-4287-9-S1-P31

**Published:** 2011-03-10

**Authors:** Mala Pande, Chris Amos, Patrick Lynch, Laura Lucio, Jinyun Chen, Marsha Frazier

**Affiliations:** 1 Department of Epidemiology, The University of Texas MD Anderson Cancer Center, Houston Texas, USA; 2 Department of Gastroenterology, Hepatology and Nutrition, The University of Texas MD Anderson Cancer Center, Houston, Texas, USA; 3 Program in Human and Molecular Genetics, The University of Texas Graduate School of Biomedical Sciences, Houston, Texas, USA

## Background

The spectrum of cancers seen in a hospital based Lynch syndrome registry was examined to compare the incidence of colorectal versus extracolonic cancers by sex, race, mismatch repair (MMR) gene mutation, age at diagnosis and index cancer. We also examined rare extracolonic cancers that are not considered to be part of Lynch syndrome.

## Method

There were a total of 463 cancers recorded in 239 patients from 150 families enrolled in the Lynch syndrome registry at MD Anderson Cancer Center. Each of these patients had a confirmed germline MMR mutation. Overall there were 218 (47.1%) colorectal and 245 (52.9%) extracolonic cancers. There was no difference in the total number of colorectal and extracolonic cancers in MLH1 mutation carriers (50.3% vs 49.7%) whereas for MSH2 and MLH6 mutation carriers there were more extracolonic than colorectal cancers overall (54.2% vs. 45.8% for MSH2 and 61.9% vs. 38.1% for MSH6). Men had fewer extracolonic cancers than colorectal (44.6% vs. 55.4%), whereas women had more extracolonic cancers than colorectal (58.9% vs. 41.1%). The mean age at diagnosis for extracolonic cancers was older than for colorectal, 48.6 vs. 44.5 years (P=<0.001). There were no significant differences by race between the colorectal and extracolonic cancers in Whites, African Americans and Hispanics.

## Results

The index cancer was colorectal in 54.8% of patients. Among the extracolonic index cancers endometrial/uterine was the most common (29.6%) followed by skin (24.1%, of which 11.1% were confirmed cases of Muir-Torre). Other index cancers were renal/ureter/bladder (10.2%), upper GI/small intestine (6.5%), ovary (5.6%) and hepatobiliary/pancreas (3.7%). It was interesting to note that the index tumor was breast cancer in 5 patients (4.6%) although breast cancer is not considered part of Lynch syndrome [1-3]. Other non-Lynch syndrome index cancers recorded were prostate (n=3, 2.8%), thyroid (n=2, 1.85%), cervix/vagina (n=2, 1.85%) and 1 case each of thymoma, leukemia, lymphoma soft palate, adenocarcinoma of the lung and fibroxanthoma peritoneum. In addition, there was 1 index brain tumor and 4 patients presented with metastatic disease (lung, liver, unknown primary).

## Conclusion

In this study, we found that Lynch syndrome patients can commonly present with cancers other than colorectal and endometrial. This has clinical relevance both for diagnosis of Lynch syndrome and surveillance for cancers of different sites during followup of these patients.
